# Data on benthic species assemblages and seafloor sediment characteristics in an offshore windfarm in the southeastern North Sea

**DOI:** 10.1016/j.dib.2022.108790

**Published:** 2022-11-29

**Authors:** Katharina Teschke, Manuela Gusky, Lars Gutow

**Affiliations:** Alfred Wegener Institute Helmholtz Centre for Polar and Marine Research, Am Handelshafen 12, Bremerhaven 27570, Germany

**Keywords:** Benthos, Biodiversity, Biomass, Abundance, Environmental impact assessment, Monitoring, Renewable energy, German Bight

## Abstract

The German Bight (North Sea) is a centre of development of offshore wind energy. In the near future, windfarms will cover a significant part (about 25%) of the German Exclusive Economic Zone. In order to understand and assess potential effects of the construction and early operational phase of offshore wind turbines on the marine environment, an extensive research programme was carried out at Germany's first offshore windfarm *alpha ventus*. Here, data are presented on macroinfauna and local sediment characteristics collected as part of this programme. Grab samples were taken annually in autumn in 2008 (baseline), 2009 (construction phase) and 2010 and 2011 (early operational phase). Sampling stations were located along transects between adjacent turbines inside the windfarm and in two reference areas with similar environmental conditions in terms of sediment characteristics and water depth. A total of 336 samples were taken inside the windfarm and 192 samples in the reference areas. Sediment characteristics were described in terms of grain size distribution and organic content. The infauna was taxonomically analysed and quantified in terms of abundance and biomass. One-hundred three infauna taxa were identified, mainly belonging to the polychaetes, crustaceans and bivalves, living in fine to medium sandy soft bottom in water depths ranging from -27 m to -30 m. The data can be useful in meta-analyses of renewable energies impacts. Additionally, the data can support species distribution modelling to gain a better understanding of species' requirements and habitats as a basis for spatial planning scenarios and the evaluation of the ecological status of the marine environment. Moreover, the data can serve as baseline data for future monitoring and management of nearby protected areas where environmental conditions are comparable to those of the present study area.

**Specifications Table**

**Table:** 

Subject	Ecology
Specific subject area	Marine benthic assemblage dynamics
Type of data	Table
How the data were acquired	Infauna and sediment samples were taken with the van Veen grab (area: 0.1 m²). Samples for infauna analysis were sieved by 1000 µm mesh size and preserved in buffered 4% formalin-seawater solution. Sediment samples were frozen. In the laboratory, taxa were identified to the lowest taxonomic level possible and their abundance and total wet mass assessed. Sedimentological analysis was performed to characterise the grain size distribution (sieve mesh sizes: 4000, 2000, 1000, 500, 250, 125, and 63 µm) and to determine the organic content as mass loss on ignition.
Data format	Raw
Description of data collection	Infauna and sediment samples were collected at four sites in the German Bight (North Sea), i.e., at two wind turbines inside the offshore windfarm *alpha ventus* and in two reference areas outside the windfarm. At each site, two transects were established perpendicular to each other, with one transect in the main current direction. Along each transect, seven sampling stations, each 100 m apart (within windfarm) and four sampling stations, each 200 m apart (within reference areas) were positioned. Three replicate samples were taken at each station to describe the infauna community. Sediment characteristics were determined from one sample per station. Sampling took place once a year in autumn from 2008 to 2011 (2008 - baseline; 2009 - windfarm construction phase; 2010 and 2011 - windfarm in operation).
Data source location	Institution: Alfred Wegener Institute, Helmholtz Centre for Polar and Marine Research, Bremerhaven, Germany Data were collected inside the German offshore windfarm *alpha ventus* and in two nearby reference areas. The sampling area extends from 53.99980 N to 54.02800 N, and from 6.537300 E to 6.679800 E. Sampling stations are displayed in Fig. 2 of this publication. The precise sampling coordinates are given with the raw data.
Data accessibility	Primary data are available on PANGAEA data repository: (1) Data on infauna: https://doi.org/10.1594/PANGAEA.943325 (2) Data on sediments: https://doi.org/10.1594/PANGAEA.943326 Creative Commons Attribution 4.0 International (CC-BY-4.0)

## Value of the Data

• The data are useful for researchers in marine ecology, species community and population dynamics, biodiversity, marine conservation and engineering. Additionally, the data are useful for authorities engaged in environmental impact assessments for renewable energies. Accordingly, the data may support approval procedures for offshore windfarm projects.
• The data provide detailed information on the development of marine benthic species and species communities before, during and after the construction of an offshore windfarm in the southeastern North Sea. Accordingly, the data can be used for a variety of scientific and administrative processes. For example, the data can promote the fundamental understanding of marine ecosystem structure and functioning under changing environmental conditions. Additionally, they can support decision making for future offshore windfarm projects in terms of site selection.
• The data can be used in meta-analyses to identify common and site-specific environmental effects of offshore windfarms [1]. An increasing number of offshore windfarms are currently being constructed in coastal and offshore waters around the world. Accompanying environmental impact assessments provide a solid database to evaluate the environmental compatibility associated with these industrial ventures.
• Beyond the specific topic of environmental impact assessments of renewable energies, the data can be considered in species and community distribution modelling. These approaches have been suggested as a useful tool for the evaluation of the environmental status of marine ecosystems according to international legislations, such as the European Marine Strategy Framework Directive [2].
• The dataset provides information on interannual variability in species abundance and biomass. This information can be essential for the development of future studies and monitoring programmes on the responses of benthic communities to environmental change, as it allows for calculating the sample size needed to achieve sufficient statistical power.
• The data provide a “historical” background on the structure and dynamics of the benthic system. This background can be essential for the evaluation of the efficiency of management measures, such as the exclusion of bottom trawling activities, which are planned in the nearby Borkum Reef Ground Marine Protected Area.

## 1 Objective

The data were collected as part of an extensive research programme to investigate the effects of offshore windfarms on the marine ecosystem and to evaluate existing concepts for mandatory environmental impact assessments related to offshore renewable energies. Within this programme, various ecosystem components were investigated, such as seabirds and migrating birds, marine mammals and the benthic system. The data presented here focus on the seafloor macroinfauna. The existing concept for environmental impact assessment for offshore windfarms was extended to (1) test for potential shortcomings of the concept and (2) specifically address the processes occurring at different distances from single wind turbines of the windfarm. The data were collected in the first German offshore windfarm *alpha ventus* in order to evaluate existing methods and concepts and to support the development of corresponding future investigations and environmental impact assessments. The data have not yet been used in an original research article.

## 2 Data Description

### 2.1 Infauna Dataset

The dataset includes point-georeferenced information about density (n/0.1 m²) and wet biomass (g/0.1 m²) of infauna taxa. Metadata inform about date and time of the sampling, geographic location (WGS 84 coordinates), sampling area, sampling gear, water depth (m) as measured on board of the research vessel, as well as grab penetration depth.

**Fig. 1:**
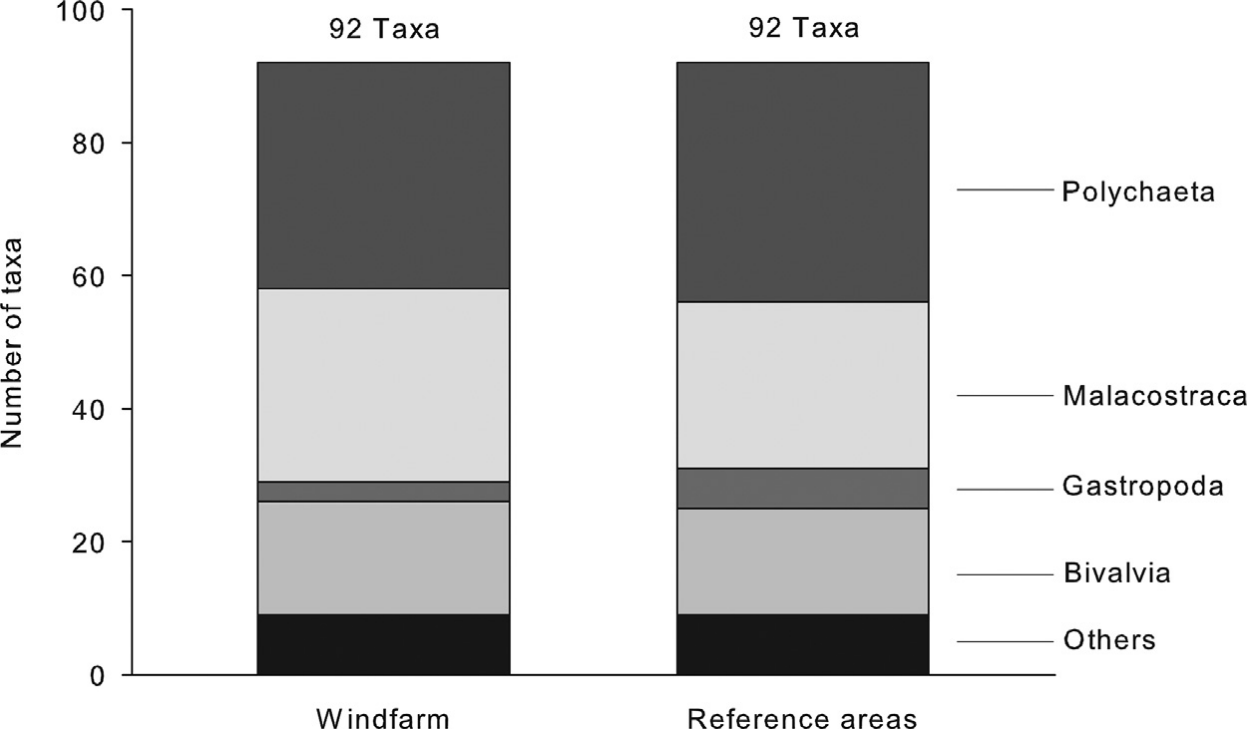
Distribution of infauna taxa among major taxonomic groups in samples collected inside the windfarm *alpha ventus* and in two reference areas in the years 2008 to 2011.

In total, 336 and 192 infauna samples were taken inside the windfarm area and in the reference areas, respectively. The infauna dataset comprises 11,399 count and 11,376 biomass records for a total of 103 infauna taxa (89% at species level, 11% at higher taxonomic levels). The taxa belong mostly to the major taxonomic groups of polychaetes, crustaceans, and bivalves, encompassing 88,370 individuals and a total biomass of 9.7 kg (Table 1; Fig. 1).

**Table 1:** Regional and annual summary of data describing the infauna communities inside the windfarm *alpha ventus* and in two reference areas.

	Windfarm				Reference areas			
Sampling year	2008	2009	2010	2011	2008	2009	2010	2011
Total taxa identified (n)	63	66	67	77	60	69	62	75
Total individuals (n)	9,835	9,227	8,372	14,679	14,119	11,651	8,504	11,984
Total biomass (g)	1,219	962	1,111	1,540	1,262	1,005	1,179	1,426

In total, 23 entries on the biomass of the sea urchin *Echinocardium cordatum*, one entry on the number of individuals of *E. cordatum* as well as one biomass entry for the amphipod *Leucothoe incisa* are missing from the dataset. Biomass values for *E. cordatum* are missing because individuals were destroyed during the sampling procedure making it impossible to properly determine the biomass and the number of individuals of this species. In these cases, the density was given as 0.5 individuals/0.1 m² by default.

### 2.2 Sediment Dataset

The dataset includes point-georeferenced information about sediment characteristics, i.e., grain size distribution and organic content (as mass loss on ignition). Metadata inform about date and time of the sampling, geographic location (WGS 84 coordinates), sampling area, sample method, and total sample mass.

Sediments were characterised for a total of 176 van Veen grab samples (windfarm: 112 grabs, reference areas: 64 grabs). The predominant grain size class of sediments was fine sand (grain size: > 125–250 µm), followed by medium sand (grain size: > 250–500 µm) in both the windfarm and reference areas (Table 2). The organic content with an average of constantly less than 1% of the total sample dry mass was low in both the windfarm and the reference areas.

**Table 2:** Regional and annual average sediment grain size distributions and sediment organic contents.

	Windfarm				Reference areas			
Sampling year	2008	2009	2010	2011	2008	2009	2010	2011
Granules (% > 2 mm)	0.1	0.1	0.2	0.2	0.1	0.1	0.2	0.3
Very coarse sand (% > 1 mm)	0.2	0.2	0.2	0.3	0.2	0.2	0.3	0.3
Coarse sand (% > 500 μm)	0.3	0.3	0.4	0.4	0.3	0.3	0.4	0.4
Medium sand (% > 250 μm)	11.3	12.0	13.7	16.8	11.4	12.3	13.7	7.2
Fine sand (% > 125 μm)	81.6	80.9	80.0	77.3	81.4	80.9	79.5	85.1
Very fine sand (% > 63 µm)	4.4	4.4	3.6	3.2	4.6	4.1	3.8	4.2
Silt (% < 63 µm)	2.3	2.1	2.1	1.9	2.2	2.2	2.2	2.6

Organic content (%)	0.51	0.46	0.47	0.46	0.47	0.48	0.50	0.63

## 3 Experimental Design, Materials and Methods

**Fig. 2:**
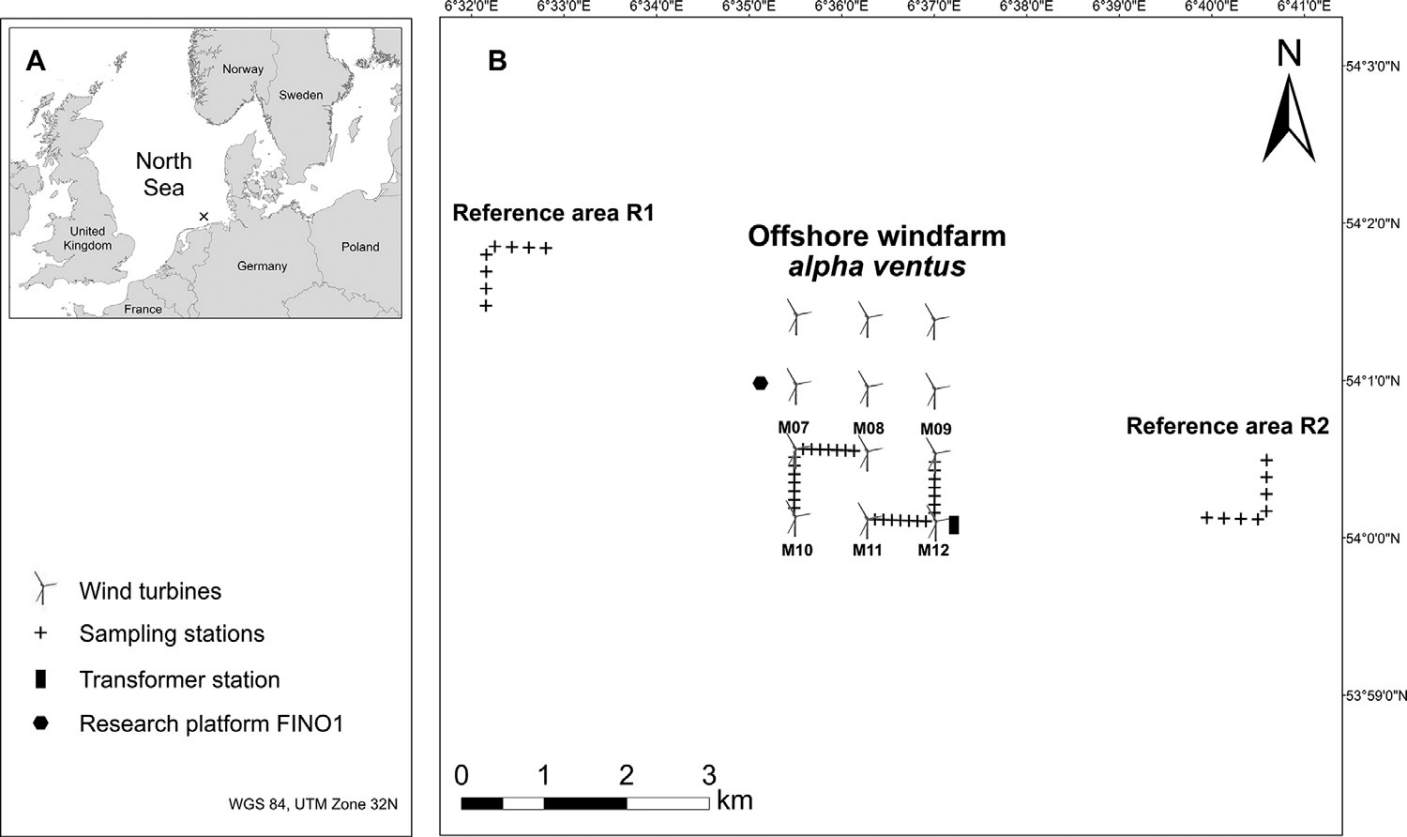
(A) Location of the offshore windfarm *alpha ventus* in the German Bight, North Sea. (B) Crosses indicate the position of the sampling stations inside the windfarm and in the western (R1) and eastern (R2) reference areas. M07 to M12 = Multibrid wind turbines 07 to 12.

The test site *alpha ventus* was the first offshore windfarm that was established in the German Exclusive Economic Zone of the North Sea. The windfarm was constructed in 2009 and commissioned in April 2010. The windfarm was composed of twelve turbines and one transformer station (Fig. 2). The turbines were arranged in a rectangular grid of four rows with three turbines each at distances of 800 m between two neighboring turbines. The windfarm comprised two types of wind turbines. The six northernmost turbines of the Repower 5M type rested on a four-legged jacket-type foundation [3]. The six southernmost turbines of the AREVA Multibrid M5000 type rested on tripod foundations. The transformer station, located in the southeastern corner of the windfarm area, rested on a jacket-type foundation. Additionally, the research platform FINO 1 was located at about 420 m west of *alpha ventus* resting on a jacket-type foundation. All underwater constructions were anchored by pillars driven 30–35 m deep into the sediment. No scour protection was deployed at the bases of the foundations. A safety zone of 500 m was established around the entire windfarm perimeter. Access to this safety zone, any activities apart from construction and maintenance works as well as research activities (summarized in Ref. [4]) were prohibited.

To investigate the effects of the wind turbine foundations on the benthic system, the infauna communities and sediment characteristics were monitored on four transects inside the windfarm and on four transects each in two reference areas outside the windfarm. Reference areas were selected with sediment characteristics and water depths similar to the windfarm area. The sediments of the windfarm area and the two reference areas were characterized as fine sand. The water depth ranged from -27 m to -29 m inside the windfarm and from -29 m to -30 m in the reference areas [5]. The reference areas were located at distances from the windfarm of 3.2 and 3.7 km, respectively.

Inside the windfarm, the transects were positioned to cover the entire distance between two neighboring turbines. The transects inside the windfarm were oriented in east-west direction between the turbines M07 and M08 and between the turbines M11 and M12 (Fig. 2). The transects extending in north-south direction were located between turbines M07 and M10 and between turbines M09 and M12. The transects extending in east-west direction were oriented along the dominant current direction, whereas the transects oriented in north-south direction were oriented perpendicular to the dominant current direction. Sampling stations along the transects were positioned 100 m apart from each other resulting in seven regularly spaced stations on each transect. In each of the two reference areas (R1 and R2; Fig. 2), two sampling transects were established, one oriented in east-west direction and one oriented in north-south direction. Along the transects in the reference areas, the sampling stations were positioned at distances of 200 m from each other resulting in four regularly spaced sampling stations on each transect at distances of 100, 300, 500 and 700 m from the starting point of the transect.

Samplings were conducted once a year in autumn (September to November) from 2008 to 2011. Samples were taken from aboard the German research vessel *Heincke* (cruise numbers: HE296, HE313, HE340, HE369). The sampling campaign of 2008 was conducted prior to the construction of the windfarm. The sampling campaign of 2009 was conducted during the construction phase, while the campaigns in 2010 and 2011 describe the conditions during the first two years of the operational phase of the windfarm.

Sampling and sample preparation in the field were carried out according to the guidelines for quantitative sampling and sample preparation of marine soft bottom macrofauna (ISO 16665, [6]). At each station, sediment samples were taken with a van Veen grab (0.1 m^2^, 90 kg, equipped with sieve lid). For the last two meters above the seafloor, the grab was lowered at a maximum speed of 0.2 m s^−1^ in order to minimize disturbance of the surface sediment and organisms by the pressure wave of the descending grab. For each sample, the geographic position was recorded as the ship position taken from the ship-based dGPS system at the moment the grab had bottom contact. Additionally, the water depth was recorded for each sample from the ship-based echo sounder. Occasionally, no water depth information was displayed by the echo sounder. Accordingly, no water depth data are available for a total of 13 samples. The penetration depth of the grab into the sediment was measured through the open sieve lid of the grab by a folding ruler to the nearest 0.5 cm. Three replicate samples were taken at each station to describe the infauna community. The samples were sieved through a metal sieve with a mesh size of 1000 µm to retrieve the organisms of the macroinfauna. Subsequently, the samples were preserved in 4% formalin-seawater solution buffered by sodium tetraborate to neutralise acidity and stored for taxonomic analysis in the laboratory.

Sample preparation in the laboratory and taxon identification and quantification were also carried out according to ISO 16665 [6]. All organisms were determined to the lowest taxonomic level possible. Single individuals of each taxon were transferred to 70% ethanol for long-term storage as taxonomic reference material. For each species, the number of individuals were determined per sample as the number of heads. Colonial species, for which the number of individuals could not be determined (e.g., hydrozoans, bryozoans) were recorded as "present" with an abundance of one individual per sample. Additionally, the total wet mass (balance model: Sartorius 1712 MP8; accuracy: 0.0001 g) per species was determined according to ISO 16665 [6].

When only fragments of solitary species were found in the sample but no heads, the abundance of this species was recorded as 0.5 individuals. Some sea urchins of the species *Echinocardium cordatum* were entirely destroyed during the sampling procedure so that it was impossible to properly determine the abundance and biomass of this species. In these cases, the abundance of this species was recorded as 0.5 individuals, but no biomass data were provided. Organisms of the epifauna are not representatively sampled with a van Veen grab. Therefore, epifauna organisms, which were occasionally found in the samples, were removed from the dataset.

Another sediment sample was taken at each station for the analysis of sediment characteristics. A subsample of 95 mL was taken from the surface of the van Veen grab sample using a corer (diameter: 4.5 cm, penetration depth: 6 cm), transferred into a labelled Ziploc bag, and stored frozen at -20 °C until analysis of the grain size distribution and organic content (according to ISO 16665 [6]). The grain size distribution was determined according to DIN18123 [7]. For the analysis of the grain size distribution, a fraction of the sub-sample was dried in a drying oven at 105 °C for 24 h and the total dry mass of the sample was measured. The dry mass of the fractions ranged from 163 to 719 mg. Subsequently, the dry sediment was fractioned by grain size in a sieve cascade with declining mesh sizes of 4000, 2000, 1000, 500, 250, 125, and 63 µm (sieving device: Retsch AS 200 basic). The dry mass of each fraction was measured (balance model: Kern KB2000-2N; accuracy: 0.01 g). The remaining fraction of the sub-sample was used to determine the organic content as mass loss on ignition according to DIN 38414-3 [8]. This sediment fraction was also dried at 105 °C for 24 h and the dry mass was measured. Subsequently, the sediment was combusted at 500 °C (annealing furnace: Linn High Therm LM 412.07) for three hours and the mass of the remaining ash content was determined. All sediment fractions and the remaining ash content were discarded after analysis. The analyses of the sediment characteristics were conducted by the Institut für angewandte Ökosystemforschung GmbH (Neu Broderstorf, Germany).

## Ethics Statements

This study does not involve animal experiments.

## CRediT authorship contribution statement

**Katharina Teschke:** Conceptualization, Data curation, Formal analysis, Investigation, Methodology, Validation, Visualization, Writing – original draft. **Manuela Gusky:** Data curation, Investigation, Methodology, Writing – review & editing. **Lars Gutow:** Conceptualization, Data curation, Formal analysis, Funding acquisition, Investigation, Methodology, Project administration, Writing – review & editing.

## Declaration of Competing Interest

The authors declare that they have no competing financial interests or personal relationships that could have appeared to influence the work reported in this paper.

## Data Availability

Count and biomass records of infauna and sediment characteristics from grab samples at the alpha ventus offshore wind farm (North Sea), 2008 - 2011 (Original data) (PANGAEA).
